# Tools and approaches to operationalize the commitment to equity, gender and human rights: towards *leaving no one behind* in the Sustainable Development Goals

**DOI:** 10.1080/16549716.2018.1463657

**Published:** 2018-05-29

**Authors:** Gerardo Zamora, Theadora Swift Koller, Rebekah Thomas, Mary Manandhar, Eva Lustigova, Adama Diop, Veronica Magar

**Affiliations:** Gender, Equity and Human Rights Team, World Health Organization, Geneva, Switzerland

**Keywords:** Health equity, gender, human rights, Sustainable Development Goals

## Abstract

The objective of this article is to present specific resources developed by the World Health Organization on equity, gender and human rights in order to support Member States in operationalizing their commitment to leave no one behind in the health Sustainable Development Goals (SDGs), and other health-related goals and targets. The resources cover: (i) health inequality monitoring; (ii) barrier analysis using mixed methods; (iii) human rights monitoring; (iv) leaving no one behind in national and subnational health sector planning; and (v) equity, gender and human rights in national health programme reviews. Examples of the application of the tools in a range of country contexts are provided for each resource.

## Background

In the years leading up to the formulation of the Sustainable Development Goals (SDGs) there was increasing awareness that the Millennium Development Goals (MDGs) insufficiently addressed equality and the reduction of inequalities [1], focused on and sought aggregated information (which some called the tyranny of national averages [2]), did not address within-country inequalities [3] and had a weak human rights approach [4]. This was noted in the United Nations General Assembly (UNGA) 2010 resolution that reviewed progress towards achieving the MDGs outcomes [5] and the UNGA 2015 resolution that established the goals of the SDGs-related targets [6]. The latter stressed tackling inequalities within and among countries, working towards gender equality, protecting human rights and addressing discrimination as core elements of the post-2015 development agenda. This was reflected in the Goals’ formulation and targets. For instance, the inclusion of the words ‘for all’ in SDG 3 and the pledge to universal health coverage in Target 3.7 reflect a commitment to equity, and is in line with international human rights obligations to prevent discrimination of any kind based on ethnicity, colour, sex, language, religion, political or other opinion, national or social origin, property, birth or other status such as disability, age, marital and family status, sexual orientation and gender identity, health status, place of residence, economic and social situation. SDG 5 and its targets address gender equality, with linked dimensions in many other Goals. SDG 10 tackles income inequalities and interlinked disparities. SDG 16 addresses social participation, accountability and non-discrimination. SDG 17 calls for enhanced national capacity on disaggregated data that allows countries to monitor inequalities. Reinforced by the United Nations (UN) action framework for equality and non-discrimination in the sustainable development agenda [7], these goals and their targets call development policymakers and practitioners to reflect a more synergistic and linked approach to inequalities, social determinants, gender and human rights as central to the SDGs agenda, including its adaptation and operationalization at country level. This is a departure from a conventional approach that tracks progress at the aggregate level to one that includes disaggregated data to identify which population groups are excluded or far worse in terms of, for instance, health outcomes and inquires on the ‘why’ behind inequalities. The transformative potential of the SDGs is also viewed with certain scepticism by those who call, for instance, for a deeper and clearer power analysis [8] or stronger human rights language [9].

**Box 1.** Integrating equity, gender and human rights into WHO’s resources for leaving no one behind: key concepts.

With respect to health, a **leaving no one behind** approach refers to vast and heterogeneous population groups, which face a wide range of barriers to health services that differ across countries, communities and individuals. As such, health system strengthening to leave no one behind needs to account for this heterogeneity and the complexity of barriers [19]. **Equity** is the absence of avoidable, unfair or remediable differences among groups of people, whether those groups are defined socially, economically, demographically or geographically or by other means of stratification. Rooted in social justice, ‘health equity’ or ’equity in health’ implies that everyone should be able to attain their full health potential and that no one should be disadvantaged from achieving this potential [41]. Health inequity is a normative concept, and thus cannot be accurately measured or monitored; however, health inequalities (i.e. observable differences between subgroups within a population) can be measured and monitored, and they serve as an indirect means of evaluating health inequity [22]. Although related, health equity and health inequalities are different from the human rights principle of *equality*, which refers to the right of every individual to receive the same treatment without discrimination. **Gender** refers to the socially constructed norms that impose and determine power, roles and relationships between groups of women, men, boys and girls in all their diversity, and which operate at various levels, from households to communities and institutions. Gender also refers to expressions and identities of women, men, boys, girls and gender-diverse people. It is important to be sensitive to different identities that do not necessarily fit into binary male or female (biological) sex categories. Gender is inextricable from other social and structural determinants shaping health and equity and can vary across time and place [41]. **The right to health**: The right to health in international human rights law is a claim to a set of social arrangements – norms, institutions, laws and an enabling environment – that can best secure the enjoyment of this right. It is an inclusive right extending not only to timely and appropriate health care, but also to the underlying determinants of health. The right to health is subject to progressive realization and acknowledges resource constraints. However, it also imposes on states various obligations, which are of immediate effect, such as the guarantee that rights will be exercised without discrimination of any kind and the obligation to take deliberate, concrete and targeted steps towards its full realization.

The mandate of the World Health Organization (WHO), as outlined in its Constitution, is to ensure the highest attainable level of health as a fundamental right for all people, a commitment that requires ensuring that no one is left behind (see Box 1), in line with the transformative approach of the 2030 Agenda. In keeping with requests from WHO Governing Bodies [10], the Organization has been providing technical assistance to Member States in a range of areas relevant to *leaving no one behind* in the SDGs.

The objective of this article is to present specific resources developed by WHO on equity, gender and human rights in order to support Member States in operationalizing their commitment to leave no one behind in the health SDGs, and other health-related goals and targets. Specifically, these resources assist Member States and their partners in addressing the root causes of social and health inequalities across the social gradient [11–14] as part of the operationalization of their SDGs commitment. Grounded in accountability, non-discrimination [15] and participatory approaches that facilitate ownership [16,17] of the processes by national participants, health authorities using these resources are accompanied in their efforts to integrate equity, gender and human rights into health policies, strategies and plans in a systematic manner. In addition, health authorities applying the resources identify the required coordination and action with other sectors with the shared aim of leaving no one behind. Also, health authorities and their partners are provided with capacity-building interventions that are designed to apply learnings once the intervention is completed, in line with an impact-oriented and resource-efficient approach [18].

**Figure 1.**
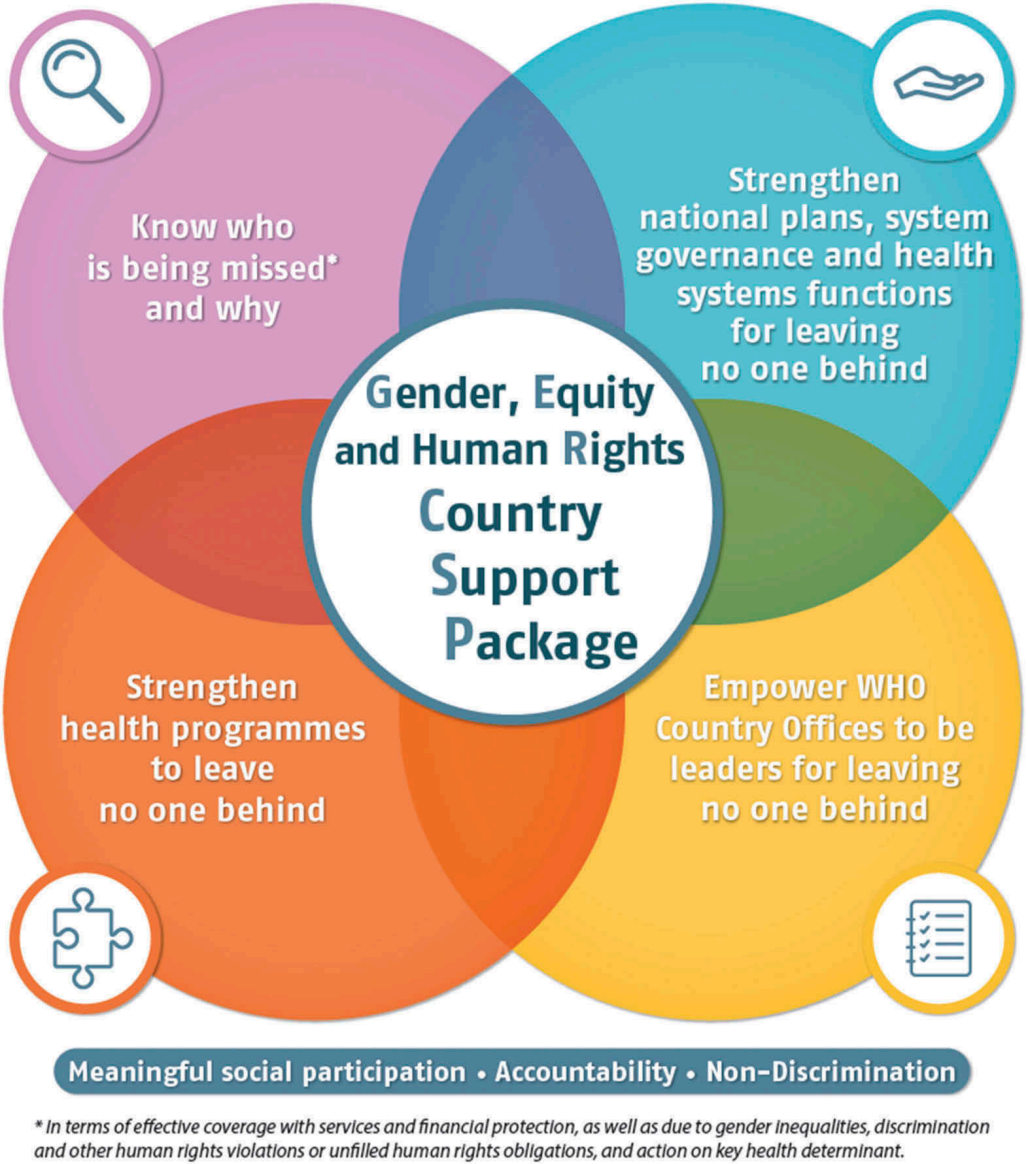
WHO’s gender, equity and human rights country support package (as of 2018).

The resources are presented to Member States as a *package* (see Figure 1) and they cover: (i) health inequality monitoring; (ii) barrier analysis using mixed methods; (iii) human rights monitoring; (iv) leaving no one behind in national and subnational health sector planning; and (v) equity, gender and human rights in national health programme reviews. A description of each component and brief examples of their application are presented next.

## Health inequality monitoring

In order to identify populations not accessing health and well-being services, as well as those faring worse in terms of determinants of health, risks and vulnerabilities, strong and reliable health information systems are necessary to conduct health inequality monitoring and they are in fact needed to support sound policy formulation and decision-making across health systems building blocks. Health data disaggregation helps governments identify the health state of the disadvantaged groups [20]. The data requires to be monitored and acted upon for addressing the root causes of inequalities through appropriate policy and programme development [21].

WHO has developed tools to provide technical support and build capacity among Member States in health inequality monitoring. The *Handbook on Health Inequality Monitoring* [22] serves as a resource to develop and strengthen inequality monitoring systems. Capacity building through training supports countries in analysing and reporting national-level data [23] . For instance, Indonesia is a country-level example for capacity-building processes to improve health inequality monitoring [24]. During 2016–2017 a network of stakeholders, led by the national authorities, committed to strengthening the country’s capacity for health inequality monitoring and that commitment, effort and process crystallized into the first-ever WHO national report on state of inequality that covers 11 health topics, drawing data from 53 health indicators and disaggregated in 8 dimensions of inequality [25]. Also, the Health Equity Assessment Toolkit Plus (HEAT Plus) was launched in July 2017. This is a software application to assess health inequalities within countries. It allows users to upload and work with any data base on any relevant equity stratifiers (e.g. sex, age, wealth status and educational attainment) [26].

These resources are part of the *Know who is being missed and why* component of the package (see Figure 1), but they can be used in conjunction with the other tools and components.

## Barrier analysis using qualitative methods

Health inequality monitoring identifies where inequalities exist and how large they are. However, addressing them requires understanding why they exist [27] . Despite the largely demonstrated social gradients in disease distribution [28], there are striking inequalities in access and use of health services affecting those who most need care [29–31]. Qualitative research methods and participatory analysis are useful for the purpose of unpacking the drivers of health inequalities. For example, key informant interviews with providers can highlight system performance deficiencies with respect to access and effective coverage of quality health services and financial protection. Focus groups can also be conducted with the target population for health interventions to identify, for example, those who complete treatment and, importantly, those who do not [27]. Upon their request, WHO has worked with national authorities and partners in different countries – including Moldova [32], Greece [33], Vietnam and Nigeria [34] – on qualitative assessments using a new tool that explores barriers from both supply and demand perspectives. In Nigeria, for example, the findings from the barrier assessment pilot in 2017 are leading to greater coordination at district level between the malaria programme and the neglected tropical diseases programme, so preventive chemotherapy becomes more available for hard-to-reach population groups. Early lessons learnt from these pilot applications have been analysed and incorporated in to the guidance tool, which is available in the public domain [34]. Further refinements will continue in the 2018–2019 biennium drawing lessons and expertise from different health domains and settings.

This resource is part of the *Know who is being missed and why* component of the package (see Figure 1).

## Human rights monitoring

To complement the qualitative barrier analysis, the international human rights monitoring mechanisms (i.e. Treaty Bodies, Special Procedures and the Universal Periodic Review) provide crucial opportunities for states and other stakeholders to review progress and challenges to realizing the right to health, against normative benchmarks of the international bill of human rights. Amongst these, the Universal Periodic Review (UPR) is a unique state-to-state peer-review process that involves a review of the human rights records of all UN Member States. Based on country-, UN- and other stakeholder-compiled information, the UPR takes place at the regular sessions of the intergovernmental Human Rights Council and provides a transparent process for states to report on actions they have taken to improve the human rights situations in their countries and what steps are being implemented to meet and fulfil their human rights obligations. The UPR enjoys unanimous support from UN Member States and reviews progress along the interdependent axes of civil, political, economic, social and cultural rights mirroring indivisibility of the SDGs and underlining their interrelatedness.

Analysis of UPR implementation in recent years has shown the extent to which health is addressed in state-issued recommendations. Nearly a quarter (22%) of recommendations from the first cycle were health-related [35]). It also shows that states are taking concrete steps to address recommendations made under the UPR, including legislative reform to end gender-based violence, ratification of international legal instruments to protect and promote health-related rights (including of migrants, women, children and sexual minorities), non-discrimination measures, and access to treatment and birth registration measures [36]. For instance, a recent non-governmental organization--led review of UPR recommendations found that 48% of them have been partially or fully implemented [37]. There is, however, still scope to make better use of these processes, and WHO is working with other UN entities and stakeholders, including the Office of the United Nations High Commissioner for Human Rights (OHCHR), to build capacity in WHO for more routine and systematic engagement with these processes with a view to advancing global health and human rights accountability and developing guidance and technical support on how to use UPR and international human rights monitoring mechanisms in WHO.

This resource is part of the *Know who is being missed and why* component of the package, but it can be used in coordination with other tools and components, especially for *empowering WHO Country Offices to be leaders for leaving no one behind* (see Figure 1).

## Leaving no one behind in national and subnational health sector planning

A critical way to influence an integrated and coherent approach to operationalizing the SDGs’ commitments to equity, gender and human rights and leaving no one behind is through National Health Policies, Strategies and Plans (NHPSPs). WHO’s guidance for NHPSPs alignment with the SDGs includes: *Strategizing National Health in the 21st Century: A Handbook* [38], and *Human Rights and Gender Equality in Health sector Strategies* [39], which focuses on practical options for policymakers to further enhance these dimensions in NHPSPs. Such guidance is used by WHO Country Offices in their work to support Member States.

Addressing demand side barriers and structural inequities such as gender inequality and other intersecting inequalities should also be addressed through social participation and community empowerment among rights holders. Using quantitative and qualitative data tools and approaches, including participatory methods with community-based groups, is necessary for improving national and subnational level health outcomes.

WHO has developed guidance for integrating a *leave no one behind* focus into subnational health planning. As requested by the country, this approach was piloted, for instance, in Mongolia in 2016–2017 with more than 100 health managers from all 21 provinces and Ulaanbaatar’s 9 districts. Participants considered how to build this focus into situation analysis and needs assessments, implementation plans, budgeting and monitoring and evaluation frameworks for subnational health sector plans [40]. For instance, access to health services by remote rural subpopulations was considered and assessed and, as a result, legal frameworks were adapted (e.g. a Ministerial Order was updated), training and capacity-building processes for health workers on *leaving no one behind* approaches were developed and implemented, and use of mobile health services in rural areas was further promoted [40].

The tool will be further piloted in 2018–2019 in a range of country contexts. This tool is part of the *Strengthen health sector planning and systems functions* component of the package (see Figure 1).

## Integrating equity, gender and human rights in national health programme reviews

WHO’s Innov8 approach [41] to reviewing national health programmes to leave no one behind provides guidance on ‘how’ to move from having data on inequalities to implementing actual changes in programmes. Made specifically for health programmes, yet underpinned with systems thinking, Innov8 is a stepwise approach to: analysing the subpopulations being missed by the programme; identifying the barriers they face; defining the potential drivers of the barriers in the health sector and beyond; and identifying what can be done to address the barriers (e.g. through changes within the health system, intersectoral action and social participation). The tool also aims to enhance health professionals’ capacities through applied learning.

The process is Government-owned with the participation of civil society organizations (CSOs). Innov8 analysis is conducted by interdisciplinary teams from the national health programme (selected by the country), other counterparts in the Ministry of Health (e.g. working on health information, planning and finance) and key stakeholders, including CSOs and district managers from the most disadvantaged parts of the country. Innov8 produces recommendations to modify the programme to ensure that needs and human rights of disadvantaged subpopulations are not overlooked and provides a method for planning how to implement these recommendations. Innov8 is designed as a reference document and supplemented by a facilitator’s manual [42].

Nepal is one of the countries where Innov8 has been applied. Under the leadership of the Ministry of Health, WHO provided support to a multidisciplinary team of public officers and CSO representatives to review the National Adolescent Sexual and Reproductive Health Programme [43]. Diverse recommendations emerged, including: (i) *Programme design*: adapt programme criteria and performance reviews for and enhance community outreach in rural remote and slum areas; (ii) *Financing*: build the economic case for investment in adolescent health and broker increased district-level funds; and (iii) *Legislation*: strengthen work across sectors to address the causes of, and enforce laws against, early marriage.

Innov8 findings were incorporated into the revised *National Adolescent Development and Health Strategy* in Nepal. A national piloting and implementation plan to take forward recommendations is being developed following endorsement of the new strategy.

The Innov8 approach has been evolving as countries apply the tool and enhance it with the implementation results (e.g. Nepal [43], the former Yugoslav Republic of Macedonia [44], Serbia [45], Indonesia [46], Morocco [47]). Innov8 is therefore conceptualized as a ‘living tool’ [41], as are all the other resources. This resource is part of the *Strengthen health programmes to leave no one behind* component of the package (see Figure 1).

## A way forward for WHO

A public health renewal, aligned with the 2030 Sustainable Development Agenda, requires us to work differently, and with new skill sets. Instead of compartmentalized modalities, equity, gender, human rights, together with social determinants approaches, must be formulated as a cohesive and collective endeavour. Research and evidence on who is being missed and why need to influence political commitment and national spheres of policymaking, programming and budgeting to ensure consistent application of data. Evidence must be generated using mixed methods and with the meaningful participation of people and communities from the start and throughout programme cycles, including community-based monitoring. This will ensure that the people’s voices are heard and are influential, that the ‘*why?*’ data are generated, and that multi-stakeholder trust and communication for accountability are enhanced. Conventional public health approaches that rely on aggregate, mainly quantitative, data and siloed project-specific interventions must be challenged and changed towards system-wide, intersectoral approaches that are value-driven.

To that end, the tools and resources presented here as a package have been co-developed with countries through participatory approaches and adapted to their context as part of the implementation. Progressive enhancement and refinement of the resources in the package is accomplished through *learning by doing* and continuous application, reflection and adaptation at the local, national and global levels with the participation of public officers, CSOs and users of health services. This approach facilitates ownership of the process by national authorities and participants. Applications of these resources have shown to effect change in health strategies, policies and programmes in various country settings and contexts with a view of improving the health of the most disadvantaged population groups. As has been presented in this paper, reducing inequalities and discrimination requires strong health systems, political commitment and social engagement. In this view, further refinements of the resources are continuously being made through incorporation of implementation results and thus support WHO Member States and their partners in operationalizing equity, gender and human rights as part of the their efforts and commitment to leave no one behind in achieving the 2030 Agenda for Sustainable Development.

## Limitations of the paper

First, while all the tools presented here have been applied in different countries, they are ‘living resources’, i.e. they are continuously revised and updated to incorporate the results of implementation, including participants’ feedback and critiques. Despite being subject to expert evaluation and reviews, an impact evaluation of the application of the tools has not been conducted at national or multinational levels. Second, these tools are being co-developed by WHO Secretariat and Member States to assist them and their partners in making the SDGs commitment to leaving no one behind operational, but the tools will not bring change unless they are accompanied by political commitment, social engagement and enhanced capacities as stated in the paper. Therefore, the tools should not be seen or used in isolation from other national and international efforts, processes and resources that also aim to bring change and support countries in achieving the SDGs. Third, the paper does not describe the types of enabling environments that are required for the uptake and application of the tools because this is beyond the purpose of this article, which is intended to present the resources and examples of application in different countries and the impacts/changes derived from the application. An analysis of support systems and enabling environments requires a different of article with information from, for instance, an impact evaluation.

## Paper context

The Millennium Development Goals (MDGs) insufficiently addressed equality and the reduction of inequalities, focused on aggregated information, did not tackle within-country inequalities and had a weak human rights approach. The formulation of the Sustainable Development Goals (SDGs) pointed out the need to fill these gaps. This paper briefly describes resources co-developed by the WHO Secretariat and Member States on equity, gender and human rights for supporting countries in operationalizing their commitment to leaving no one behind in the health SDGs, and other health-related goals and targets.
